# General versus site specific quantile random forest models for virtual patient‐specific quality assurance of VMAT plans

**DOI:** 10.1002/acm2.70854

**Published:** 2026-10-16

**Authors:** Naila Rahman, Greg Kaliel, Mark D'Souza, Dylan Yamabe Breitkreutz

**Affiliations:** ^1^ Department of Medical Physics Odette Cancer Center Sunnybrook Health Sciences Center Toronto Ontario Canada; ^2^ Department of Radiation Oncology University of Toronto Toronto Ontario Canada; ^3^ Department of Medical Physics Toronto Metropolitan University Toronto Ontario Canada

**Keywords:** machine learning, virtual patient‐specific quality assurance, VMAT

## Abstract

**Background:**

Modern radiotherapy techniques like volumetric‐modulated arc therapy (VMAT) require labor‐intensive patient‐specific quality assurance (PSQA) measurements, which for busy clinics pose a substantial workflow burden. Most of these measurements simply confirm acceptable plans. This inefficiency has motivated the development of machine learning (ML) models for virtual PSQA to predict the gamma passing rate (GPR) and safely reduce the need for pre‐treatment measurements. Plan complexity, often quantified by metrics like the small aperture score (SAS) and average leaf pair opening (ALPO), is a key predictor of PSQA outcome, and its relationship to GPR can vary across different treatment sites. Previous literature exploring ML for PSQA has focused on general models trained on a pooled dataset of all disease sites.

**Purpose:**

To rigorously compare the predictive performance of a general ML model trained on a pooled dataset of multiple disease sites against site‐specific ML models. We hypothesized that isolating plan complexity effects within each disease site would improve predictive accuracy, specifically by reducing false positives and improving specificity.

**Methods:**

A total of 15 000 VMAT PSQA measurements (3%/3 mm global criteria, ArcCheck) from multiple disease sites were retrospectively collected. We developed and compared three general models—an XGBoost regressor (XGB), a standard random forest regressor (RFR), and a quantile random forest regressor (QRF)—using 14 plan complexity features as input. Five separate QRF models were also trained for the largest site groups (GI, GU, HN, Lung, and Prostate). For clinical decision‐making, the QRF lower‐quantile estimate was used as the predicted GPR to ensure a conservative safety margin. Model performance was evaluated using mean absolute error (MAE) and clinical metrics, including sensitivity (correctly identifying passes) and specificity (correctly identifying fails), across various pass/fail thresholds.

**Results:**

The general QRF model achieved high accuracy (MAE: 0.92%), on par with measurement repeatability. The site‐specific QRF models achieved prediction accuracy comparable to or better than the general model. Importantly, they substantially improved specificity and reduced false positives. For example, at the 95% threshold, the general model had an 8.8% false‐positive rate, while the prostate‐specific and GU‐specific models produced (no false positives on the test set (specificity 100%, 19/19 and 5/5 respectively). Because the number of truly failing plans per site is small, these point estimates carry wide 95% confidence intervals (e.g., 82.4%–100% for prostate and 47.8%–100% for GU; Clopper–Pearson). Using a conservative flagged mode, site‐specific QRF models reduced false positives to near zero at the cost of reduced but clinically acceptable sensitivity (∼50%–70%). Learning curves showed that the general QRF model plateaued after ∼964 cases, but some site‐specific models, like the GU model, required less than 100 cases to reach a stable MAE.

**Conclusions:**

Tailoring virtual PSQA prediction models to specific disease sites substantially reduces false‐positive predictions without compromising overall accuracy. The high specificity of site‐specific QRF models strengthens confidence in virtual PSQA and supports clinical implementation that could reduce the number of required QA measurements. The QRF model also provides uncertainty estimates and is highly interpretable, making it a robust tool for enhancing clinical QA efficiency.

## 1 INTRODUCTION

Modern radiotherapy techniques like volumetric‐modulated arc therapy (VMAT) demand rigorous patient‐specific quality assurance (PSQA) to ensure delivery accuracy. This typically involves pre‐treatment measurements with detector arrays for each treatment plan. While effective, this process is labor‐intensive and time‐consuming for busy clinics. In practice, the yield of detecting true delivery errors is very low—for example, in a recent multi‐institution study of 5522 VMAT plans, fewer than 1% of plans failed a 95% gamma passing rate criterion.^1^ Such findings underscore that the vast majority of PSQA measurements simply confirm acceptable plans, making PSQA a substantial workflow burden. This inefficiency has motivated the development of predictive models that can forecast PSQA outcomes and thereby streamline the QA process.^2^, ^3^, ^4^ Over the past decade, numerous groups have explored “virtual PSQA” approaches in which machine learning (ML) models predict the gamma passing rate (GPR) or identify likely failures, allowing clinics to safely reduce the number of measurements.^1^, ^2^, ^3^, ^4^ Early proof‐of‐concept was provided by Valdes *et al.* with a model to virtually predict IMRT QA results,^2^ and subsequent studies have validated ML‐based PSQA in both multi‐institutional settings^1^, ^5^ and prospective clinical trials.^4^, ^6^ However, despite this progress, adoption remains limited, which highlights the need for continued validation and optimization of these approaches before they become standard of care.

A wide range of ML techniques have been applied to PSQA outcome prediction. Valdes *et al.*’s initial model employed a penalized Poisson regression (lasso) to predict gamma pass rates from plan parameters.^7^ Other early work used traditional classifiers: for instance, Granville *et al.* demonstrated that a support vector machine (SVM) could classify VMAT plans as likely passing or failing with high accuracy.^3^ Ensemble tree methods such as random forests have likewise shown promise.^8^, ^9^, ^10^, ^11^, ^12^ In parallel, researchers have investigated deep learning for PSQA. Tomori *et al.* trained a convolutional neural network (CNN) on planar dose distributions to predict gamma passing outcomes, achieving mean errors on the order of 1%,^13^ while Interian *et al.* trained a CNN on fluence maps.^14^ Similarly, others explored neural‐network models to both predict GPR values and classify QA results with good accuracy.^15^, ^16^ More recently, advanced architectures have emerged: Wang *et al.* introduced an autoencoder model capable of simultaneous GPR regression and pass/fail classification,^17^ which was later implemented in a multi‐institution clinical workflow.^5^ Matsuura *et al.* developed a generative adversarial network (GAN) to synthesize gamma dose difference distributions, enabling highly accurate GPR predictions (∼1% error) while also pinpointing failing regions.^18^ These studies collectively report that ML models can reproduce measurement‐based QA results across diverse techniques (portal dosimetry, ArcCheck, etc.), with many reporting sensitivity/specificity metrics suitable for clinical use.

One key factor in PSQA outcome prediction is plan complexity. It is well established that highly modulated treatment plans exhibit lower gamma passing rates due to increased dosimetric and delivery complexity, and a variety of plan complexity metrics have been explored to quantify this relationship. Notably, the small aperture score (SAS)—the fraction of leaf openings below a given width—and the average leaf pair opening (ALPO) are strong predictors of GPR. Several studies have identified SAS and ALPO among the top features influencing gamma pass rates.^1^, ^2^, ^4^, ^8^ For example, a higher proportion of narrow MLC apertures (high SAS) tends to correlate with lower passing rates, as complex fluence patterns are less forgiving to delivery errors. Many ML‐based QA models explicitly include such complexity indices as input features to improve their predictions.^1^, ^2^, ^3^, ^4^, ^8^ Furthermore, treatment sites inherently affect typical plan complexity and thus QA outcomes.^2^, ^19^ Sites like head‐and‐neck often require more intricate modulation than gynecological treatments, leading to systematically lower gamma passing rates on average for the former. Accordingly, disease site has emerged as an important predictor in some models: Wall and Fontenot found that treatment site was the third most influential feature in their PSQA outcome model.^8^ This suggests that a one‐size‐fits‐all prediction model may be suboptimal if it ignores site‐specific characteristics. A model trained on a mix of different disease sites might exhibit reduced accuracy or more false alarms for certain sites, due to inter‐site variability.

To address this issue, it has been proposed that QA prediction models be tailored to specific treatment sites to capture their unique plan complexity patterns.^2^, ^3^, ^8^ Until now, however, no study has rigorously compared the performance of a general all‐site model versus site‐specific models on a large scale. The present work fills this gap by developing a quantile random forest (QRF) model for VMAT PSQA outcome prediction and evaluating it under two scenarios: a single generalized model trained on a pooled dataset of multiple disease sites, and separate QRF models trained individually for specific sites. Our aim is to determine whether site‐specific modeling can improve predictive accuracy—particularly by reducing false positives and improving specificity—relative to a general model. We hypothesize that by isolating the effects of plan complexity within each disease site, the site‐specific models will better account for the nuances of each treatment site's planning characteristics, thereby providing more reliable virtual PSQA predictions. This investigation, leveraging around 15 000 ArcCheck measurement results across several sites, is the first direct comparison of general versus site‐specific ML models for PSQA prediction. Our findings will inform the optimal strategy for deploying ML‐based PSQA in clinical workflows, with the goal of maximizing efficiency while maintaining patient safety through accurate error detection.

## 2 METHODS

### 2.1 Data collection

A total of 15 076 PSQA measurements were retrospectively collected from clinical VMAT plans delivered at our institution on Elekta Agility linacs between 2013 and 2024. All planning was done on Pinnacle, including versions v9.0, v9.2, and v9.8. All PSQA measurements were performed with an ArcCheck diode array (Sun Nuclear, Melbourne, FL), following departmental protocols. Plans encompassed multiple disease sites, including gastrointestinal (GI, *n *= 1592), genitourinary (GU, *n *= 654), head‐and‐neck (HN, *n *= 2197), lung (*n *= 2250), and prostate (*n *= 2378). There were 4142 plans in the ‘Other’ site category, which involved cases such as lymphoma, chest wall, bile ducts, and overlapping lesions (lesions crossing boundaries between adjacent anatomic sub‐sites). Gamma passing rates (GPRs) were calculated using a 3%/3 mm global dose‐difference/distance‐to‐agreement criterion with a 10% dose threshold using the Van Dyk method.  The six named categories above (GI, GU, HN, lung, prostate, and Other) sum to 13 213 plans. The remaining 1863 of the 15 076 collected measurements correspond to cases that were excluded prior to modeling and were plans associated with measurement or setup errors that were subsequently re‐measured (described below). The 15 076 figure therefore refers to all measurements collected, whereas the per‐site counts and all model training and testing reflect the 13 213 plans retained after exclusions. We acknowledge that data were acquired over an 11‐year interval during which several factors could have changed, including planning policies, dose‐calculation settings, linac performance, and clinical protocols. Throughout this period the department maintained a stable PSQA workflow: two ArcCheck devices and a single gamma analysis software platform and consistent departmental measurement protocols, a fixed 3%/3 mm global gamma analysis with a 10% dose threshold (Van Dyk method). Dose calibration was performed daily on the ArcChecks, and more extensive ArcCheck array calibration was assessed annually and recalibrated when needed, ensuring the quality of gamma analyses. The three Pinnacle versions (v9.0, v9.2, v9.8) used the same collapsed‐cone convolution dose engine and beam models; incremental optimizer updates across versions are reflected in the plan complexity features used as model inputs, so their effects are at least partially captured by the model. Linacs were maintained under our routine QA program, with no MLC hardware changes (only data from Elekta Agility models were considered). We did not find any effect of temporal drift in the measured or predicted GPR as further shown in the Results and Discussion (Figures S1 and S2), and we treat the dataset as a single population for modeling.

### 2.2 Model features

14 features that may influence plan complexity were input into the model and included: total monitor units (MU), total dose, MU factor, average leaf pair opening (ALPO), irregularity, small aperture scores (SAS) at 10 and 20 mm, maximum and minimum aperture area, maximum area change, average number of control points, average gantry rotation range, gantry angle per control point, and beam energy (encoded as binary, 6X = 1). In‐house software was developed to extract the complexity features from each VMAT DICOM RT Plan file and aperture metrics were calculated for each patient plan and linked to their PSQA results. The selected complexity features are consistent with those frequently cited in the literature and have been shown to reliably predict QA outcomes.^1^, ^2^, ^3^, ^4^, ^8^ Cases associated with measurement or setup errors that were subsequently re‐measured were removed to prevent them from influencing model training.

### 2.3 Model development

We evaluated three predictive models using the same training data and features: an XGBoost regressor (XGB), a standard random forest regressor (RFR), and a quantile random forest regressor (QRF). All three models were implemented in Python using the Scikit‐learn framework and compatible libraries, which provided standardized tools for model training, hyperparameter tuning, and performance evaluation. We chose to model PSQA outcomes using regression to capture all available quantitative information, then explore whether those predictions could also support clinical pass/fail decisions. The XGB and RFR models were chosen as they are two of the most commonly applied models for PSQA outcome prediction^1^, ^8^, ^9^, ^10^, ^12^, ^20^: XGB was selected for its flexibility, scalability, and built‐in feature importance analysis, while RFR was included for its robustness, interpretability, and resistance to overfitting through bootstrapped sampling and random feature selection. The QRF model is an extension of the RFR model that outputs a prediction interval (via its quantiles) allowing some insight into prediction uncertainty. The lower and upper quantiles were set to 2.5 and 97.5, respectively. The 2.5th and 97.5th percentiles were chosen a priori to correspond to a conventional symmetric 95% prediction interval rather than being optimized against the sensitivity/specificity endpoints; we deliberately avoided tuning the quantile level on the test data to prevent optimistic bias. The 2.5th percentile thus represents a conservative lower bound on the predicted GPR. We note that the choice of lower‐quantile level is effectively a tunable operating point: a higher percentile (e.g., the 5th or 10th) would shift predictions upward, increasing sensitivity at the expense of specificity, whereas a lower percentile would do the reverse. A formal optimization of this operating point against site‐specific cost trade‐offs is a natural direction for future work. For more in‐depth analyses, we utilized the lower quantile from QRF as the predicted GPR value to err on the side of caution. This underestimation of GPR serves to minimize missed failures (false positives). Specific hyperparameters chosen for each model are included in Table S1.

Separate models were trained under two scenarios:

1. **General model** (trained with XGB, RFR, and QRF models): trained using the pooled dataset of all disease sites.
2. **Site‐specific models** (trained with only QRF models): five separate QRF models were independently trained for the largest site groups (GI, GU, HN, lung, prostate).

### 2.4 Training and validation

Data were randomly partitioned into training (80%) and independent testing (20%) subsets. Model development employed 5‐fold cross‐validation on the training set to tune hyperparameters and assess stability. Learning curves were constructed by iteratively increasing the number of training samples to determine the minimum dataset size required to maintain performance.

### 2.5 Evaluation metrics

Model accuracy was quantified using mean absolute error (MAE) and clinical performance was assessed using four different thresholds: 95–95, 93–93, 90–90, and 90–95. Here, the first number indicates the model's predicted GPR cutoff for pass/fail, and the second number indicates the measured GPR cutoff for pass/fail. For example, “95–95” means that a plan is considered a true failure if its measured GPR is below 95% (the reference/ground‐truth label) and is predicted to fail if its predicted GPR is below 95% (the model decision), whereas “90–95” pairs a measured‐failure label of < 95% with a model decision threshold of < 90%. **The first number defines the model's action threshold that would trigger a physical measurement, and the second number defines the clinical measurement pass threshold**. In the intended clinical workflow, the predicted GPR (the QRF lower‐quantile estimate) is compared against the action threshold: plans predicted to pass are candidates to have their physical PSQA measurement omitted, while plans predicted to fail—or flagged by the safety criteria below—are sent for measurement. Of the combinations evaluated, the 95–95 rule (model and measurement threshold both at the clinical 95% gamma criterion) is the pre‐specified primary decision rule we would adopt in practice, because it matches our departmental pass/fail criterion. The 90–95, 93–93, and 90–90 combinations are reported as sensitivity analyses to show how performance changes as the measurement ground‐truth and model action thresholds are relaxed or decoupled. We emphasize that, because false positives (a plan predicted to pass that truly fails) are the safety‐critical errors, the operating point is chosen to prioritize specificity, and any plan flagged by the model or the criteria below is measured. Sensitivity (the model's ability to correctly identify passing plans), specificity (the model's ability to correctly identify failed plans), true/false positive rates, and true/false negative rates were calculated for each threshold:

$$\begin{array}{c} \mathrm{Sensitivity}=\mathrm{TP}/\left(\mathrm{TP}+\mathrm{FN}\right)\\ \mathrm{Specificity}=\mathrm{TN}/\left(\mathrm{TN}+\mathrm{FP}\right) \end{array}$$

To further examine a “safe” operating mode and reduce false positives, we also evaluated flagging criteria. Plans were flagged as a ‘failing’ plan if they met any one of the following criteria: GPR predicted as < the threshold, ALPO < 10, Total MU ≥ 2500, or MU Factor ≥ 10. These numerical cutoffs were derived from both retrospective empirical observation and clinical physics experience rather than taken from prior literature and were intended as a conservative safety net to catch high‐complexity outliers that the regression might otherwise predict to pass. ALPO, total MU, and MU factor were selected because ALPO and aperture‐complexity metrics were consistently among the strongest predictors of GPR across all three models (Figure 3), and total MU and MU factor are simple, monotonic surrogates for overall modulation that are trivial to compute at plan‐review time. The specific thresholds correspond to the extreme tails of the observed feature distributions in the training data. We acknowledge that these cutoffs are institution‐specific and would need to be re‐derived from local data at another center; this is noted in the Limitations section.

## 3 RESULTS

### 3.1 Temporal drift

Because the dataset spanned 2013–2024, we assessed whether temporal drift could confound the pooled analysis. As depicted in Figure S1, neither the measured GPR nor the lower‐quantile prediction changed meaningfully over time (linear trends −0.001%/year, *p* = 0.89, and −0.009%/year, *p* = 0.40, respectively), and plan complexity as captured by ALPO was similarly stable (+0.008 mm/year, *p* = 0.89). Stratifying by disease site, no individual site showed a significant temporal trend in measured GPR, with every site‐specific slope confidence interval including zero (Figure S2), and a site‐by‐year interaction test was non‐significant for all variables (all *p* > 0.69). Temporal stability therefore held both within each site and across the cohort, supporting its treatment as a single population.

### 3.2 Site distribution, feature importance, and model performance

Figure 1 illustrates site distributions for the general, GI, and GU models. Figure 2 illustrates that all sites’ measured GPRs cluster near high passing rates. This confirms that true QA failures were rare in our data—a small fraction of plans had measured GPR under 95%. The general model's task was therefore to identify these rare failing cases without erroneously flagging the many passing cases.  Only about 5.7% of test‐set plans truly failed at the 95% criterion, so the dataset is severely class‐imbalanced. This imbalance has two important consequences for how the results should be read. First, a trivial “always‐pass” classifier—one that predicts every plan to pass—would by construction achieve 100% sensitivity and 0% specificity, and an overall accuracy of roughly 94% simply by never flagging a failure. Overall accuracy and sensitivity are therefore weak discriminators in this setting, and the meaningful contribution of the QRF models lies almost entirely in their specificity, that is, their ability to correctly identify the rare failing plans without sacrificing an unacceptable number of true passes. We accordingly report the always‐pass baseline (sensitivity 100%, specificity 0%) as a reference point against which the QRF specificity gains should be judged. Second, because the GPR distribution is so narrow and skewed toward 100%, a low MAE is not, on its own, evidence of clinically meaningful prediction: a model that simply regressed toward the population mean of ∼98%–99% would also attain a small MAE while being useless for failure detection. The MAE values reported here should thus be interpreted alongside the threshold‐based sensitivity/specificity metrics, which directly measure failure detection, rather than as a stand‐alone measure of clinical utility.

**FIGURE 1:**
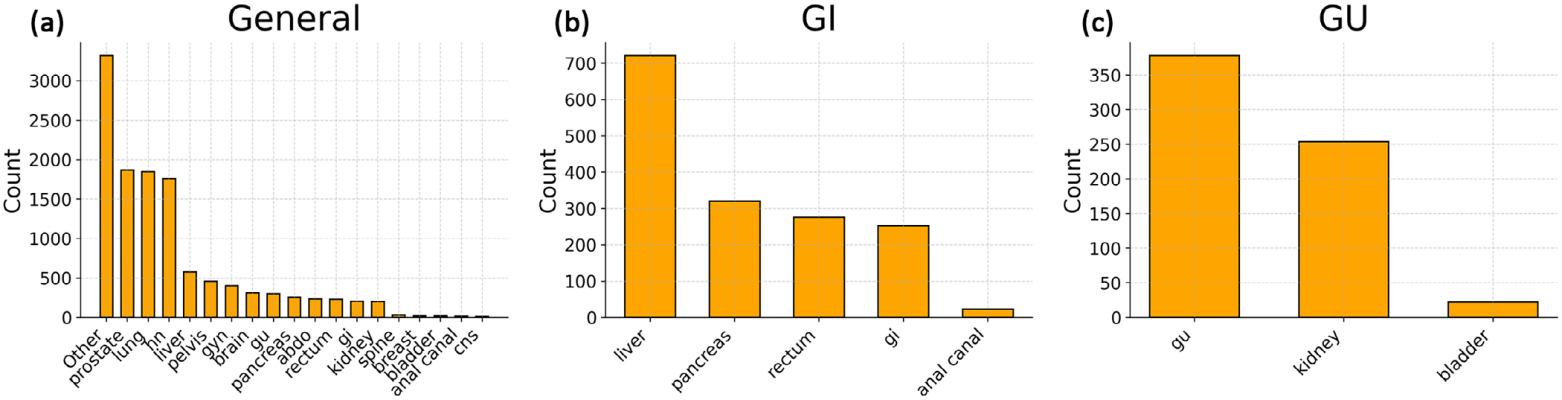
Distribution of PSQA cases across disease sites for aggregated training and testing data. (a) General: Overall counts for all sites. (b) GI: Breakdown of gastrointestinal sites, where liver cases are most frequent, followed by pancreas, rectum, gi (any other GI sites), and anal canal. (c) GU: Genitourinary cases, with gu (GU cases other than kidney, bladder, and prostate) and kidney cases forming the majority and bladder contributing only a small fraction.

**FIGURE 2:**
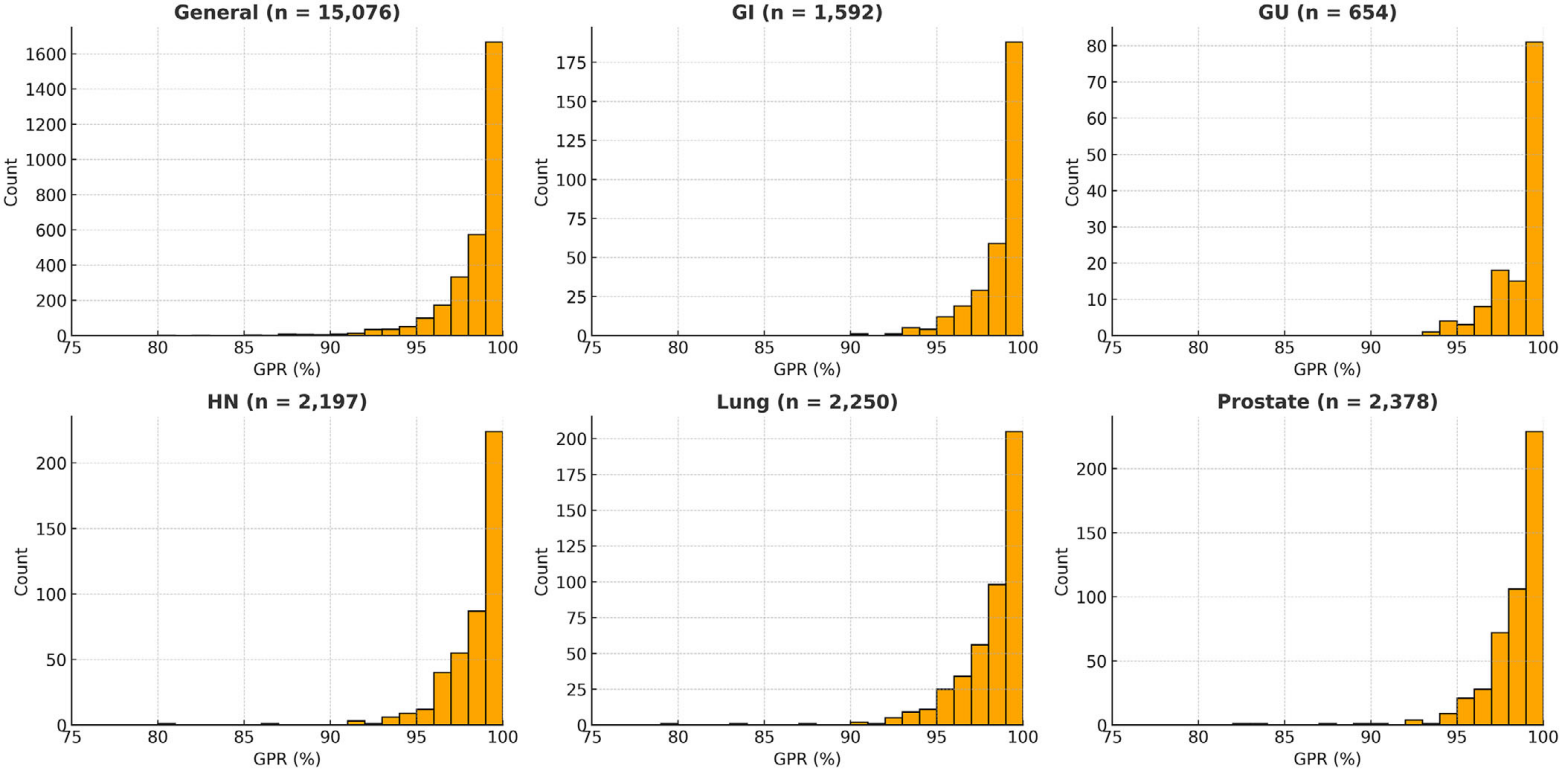
Distribution of measured GPR values for all VMAT plans in the dataset (3%/3 mm criteria). Most plans achieved high passing rates (peaking near 100% GPR), and relatively few plans fell below common clinical thresholds (e.g., 95% GPR).

All three models independently identified **small aperture score (SAS)** at 20 mm, **average leaf pair opening (ALPO)**, and **SAS at 10 mm** as the most influential features for predicting GPR (Figure 3). These features are metrics of plan aperture complexity—smaller MLC apertures and narrower openings tend to increase plan complexity and lower the gamma passing rate. The consistency of feature rankings across models indicates a robust finding: aperture size metrics dominate the prediction of QA outcomes, whereas other inputs (e.g., energy, dose, etc.) contribute less.

**FIGURE 3:**
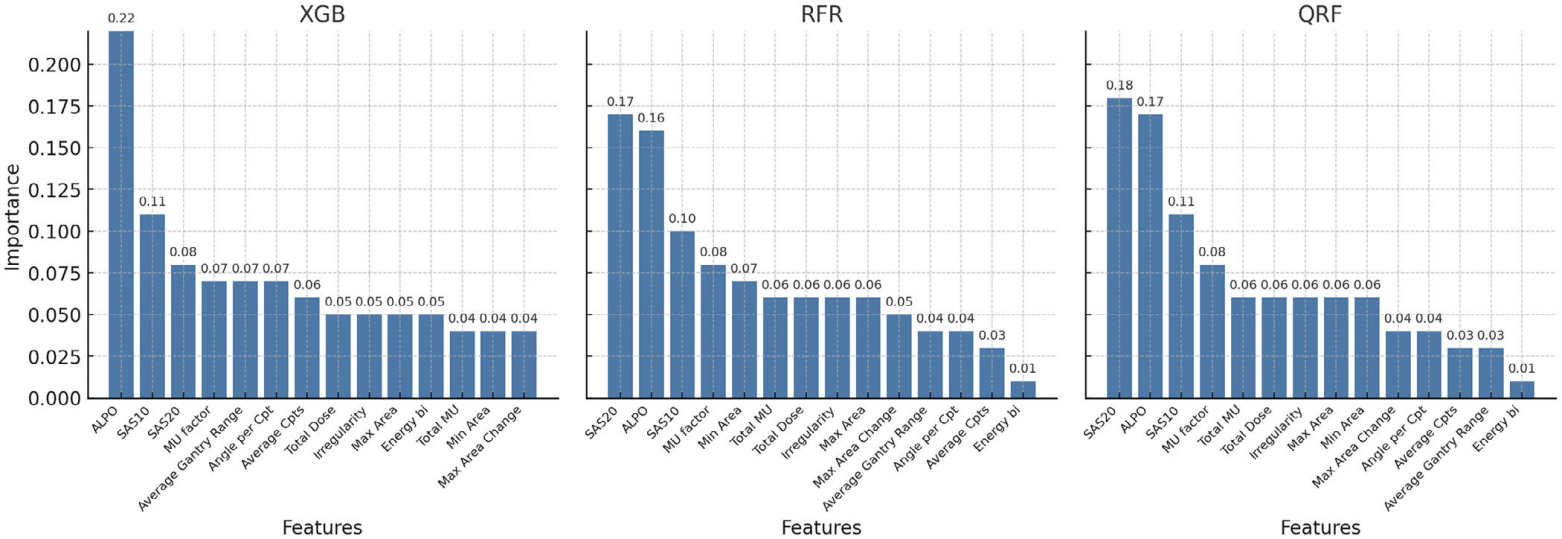
Feature importance ranking for the three machine learning models: extreme gradient boosting (XGB), random forest regression (RFR), and quantile random forest (QRF). Each model's 14 predictive features are shown in order of decreasing importance (normalized importance scores are indicated by bar lengths).

All three models, which were trained on approximately 15 000 plans spanning multiple disease sites, achieved a mean absolute error (MAE) below 1% on the independent test set (Figure 4a–c). This indicates that on average the model's GPR predictions were within 1% of the measured QA result, which is on par with measurement repeatability.^21^ For XGB (Figure 4a), **94.6%** of predicted GPRs are within ± 3% error, and **98.5%** of predictions are within ± 5% error. RFR (Figure 4b) shows similar performance with **95.1%** of predicted GPRs within ± 3%, and **98.6%** within ± 5%, highlighting excellent agreement with measurements. The QRF (median prediction) predicts **94.6% within **± 3% error, and **98.2%** within ± 5% (Figure 4c). Notably, when using the median QRF predictions, over 93% of predicted GPR values fell within ± 3 % of the measured GPR for both the general and site‐specific models, reflecting high overall prediction accuracy. As is typical of tree‐ensemble regressors, the scatter plots in Figure 4 also exhibit a mild regression‐to‐the‐mean (compression) effect, in which the highest measured GPRs are slightly under‐predicted (points falling just below the diagonal) and the lowest measured GPRs are slightly over‐predicted (points just above the diagonal). This behavior is expected because individual trees average the targets of neighboring training samples and therefore shrink predictions toward the densely populated, high‐GPR region of the data. In our case the effect is small and does not materially affect classification performance, since the lower quantile estimate already shifts predictions conservatively and the pass/fail decision depends on the threshold rather than on exact reproduction of the most extreme values. As expected, the lower quantile QRF predictions for the general model (Figure 4d) result in lower predicting accuracy with **55.6%** of predictions within ± 3%, and **81.2%** within ± 5%. This is also reflected in the higher MAE of 3.4%, as using the lower quantile predictions would result in a negative bias in the predicted GPRs. It should be emphasized that MAE is not an appropriate accuracy metric for the lower‐quantile prediction. Unlike the median prediction, the 2.5th‐percentile estimate is intentionally biased downward to provide a conservative safety margin, so its “error” relative to the measured GPR largely reflects this deliberate offset rather than a deficiency in predictive quality. The 3.4% value is reported only to characterize the magnitude of the conservative shift and should not be compared directly with the ∼0.92–0.98% MAEs of the median and point‐estimate models; the lower‐quantile predictions are instead evaluated through the threshold‐based sensitivity and specificity metrics for which they are designed. Importantly, for the 95–95 threshold, the median QRF predictions correctly identified 99.6 % of measured passing plans, but only 14 % of truly failing plans were predicted as a fail. Although the lower quantile QRF predictions correctly identified a lower percentage of truly passing plans, about 70.3% of true passing plans, there is a dramatic increase in correctly identified true failing plans (91.2%).

**FIGURE 4:**
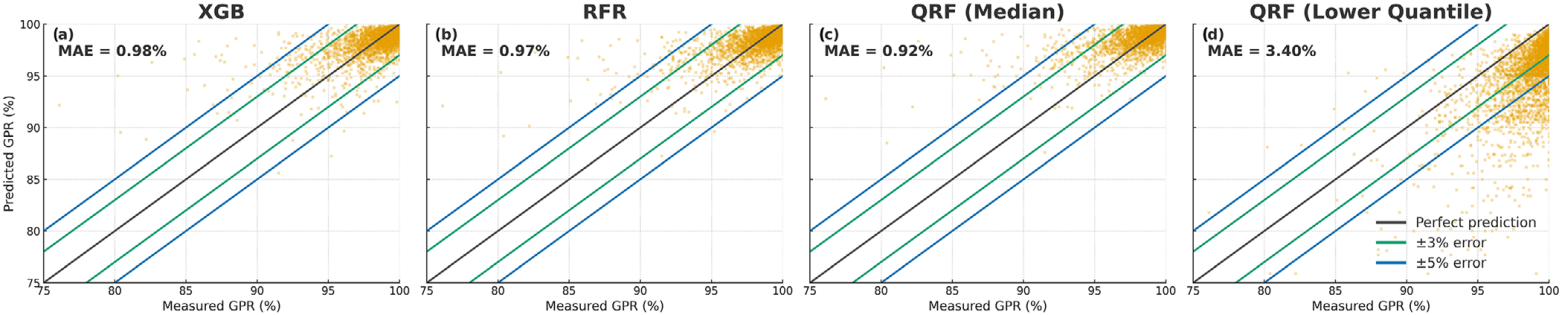
Distribution of true (measured) and predicted gamma passing rate (GPR) for the XGB (a), RFR (b), and QRF (c and d) general models, which include all sites. All plots include the 3016 cases in the testing dataset. Panel (c) shows the median predictions from the QRF model and panel (d) shows the lower quantile predictions from the QRF model.

Each site‐specific model achieved prediction accuracy comparable to or better than the general model, except for HN (Figure S3). The genitourinary (GU) site model was especially precise, with *all* median QRF predictions within 5% of the measured GPR, while the other site‐specific models had only 1.5%–2.5% predictions (median QRF) differing by more than 5% from the measurement. These results suggest that modeling each disease site separately can capture the nuances of plan complexity specific to that site, yielding very tight agreement with measured QA outcomes. Site‐specific distributions for lower quantile QRF predictions reveal the same trends as median predictions, but with higher errors, as expected. Figure S4 shows that the lower‐quantile GPR predictions are markedly more variable than the median predictions, highlighting prediction uncertainty. Across sites, ∼40%–60% of median predictions fall in the 99%–100% GPR bin, whereas < 5% of lower‐quantile predictions do.

Accordingly, the QRF lower‐quantile estimate was used as the predicted GPR for all analyses. This choice provides a conservative safety margin and is expected to reduce false positives. **Unless noted otherwise, ‘predicted GPR’ hereafter refers to the QRF lower‐quantile estimate**.

Sensitivity and specificity metrics reveal the QRF model attained the highest specificity (91% for the 95–95 threshold) among the three models (Figure 5). The XGB and RFR models show substantially lower specificity (25%–29%), indicating a substantially higher false‐positive rate compared to QRF (70%–75% compared to 8% at 95–95). Sensitivities were exceptional for XGB and RFR (98%–100%) and moderate for QRF (67%–82%). Overall, QRF provided the best balance, especially by keeping the false‐positive rate to a minimum. The slight trade‐off is a reduction in sensitivity; however, QRF's sensitivity remained comparable to other models in literature^1^, ^4^, ^6^, ^18^ and acceptable for clinical use.

**FIGURE 5:**
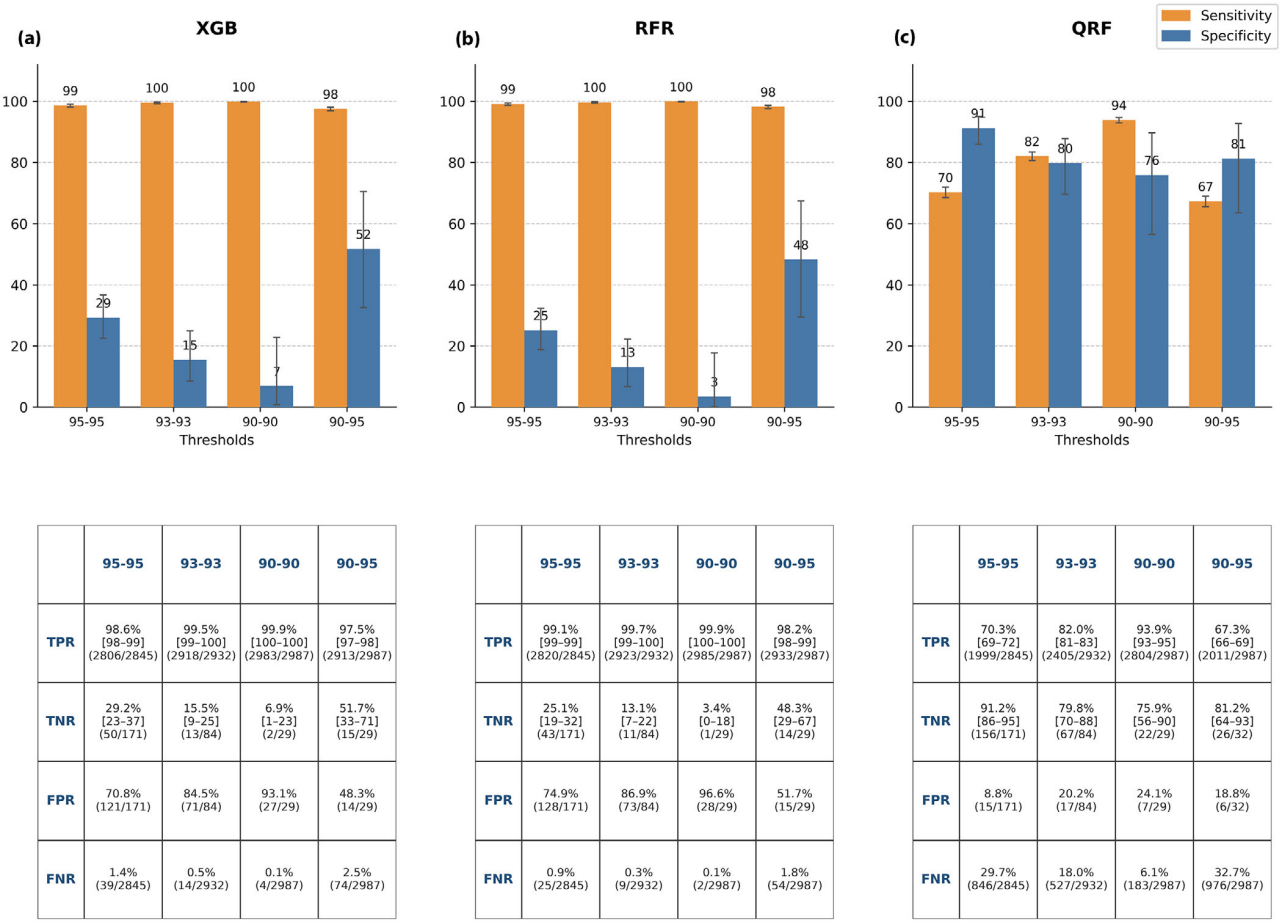
Sensitivity and specificity achieved by each general model (XGB, RFR, QRF) at the various thresholds. True/false positive rates (TPR and FPR respectively) and true/false negative rates (TNR and FNR respectively) are reported in the tables. Sensitivity refers to the TPR and specificity refers to the TNR. Sensitivity (TPR) and specificity (TNR) are each accompanied by their 95% Clopper–Pearson confidence intervals (in square brackets beneath the point estimate and count), with matching error bars on the bars.

### 3.3 Site‐specific sensitivity and specificity

Figures 6 and 7 summarize the QRF model outcomes for general versus site‐specific models, under two different modes—with no flagging criteria (Figure 6) and using flagging criteria (Figure 7). In Figure 6, a plan is predicted to pass only if the predicted GPR is above the clinical threshold (e.g., 95%). Under this condition (no extra safety margin), all models already exhibit high specificity and sensitivity, but the site‐specific models demonstrate clear advantages in certain cases. For example, at 95–95 threshold the general model produced 15 false positives, whereas the GU‐specific and prostate‐specific QRF models produced 0 false positives, and the lung model only 1 false positive. The HN model had 4 false positives, which was higher than other sites but still low in absolute count. In the general model, 40% of the 17 false positives (out of 3016) that were detected using a 93–93 threshold were HN cases, and 3 false positives were detected using 90–95. All site‐specific models achieved specificities above 80%, with GU and prostate reaching 100% specificity (no false positives at all). The cost of this high specificity was a slight drop in sensitivity for some site models. Nonetheless, for all site models the sensitivity remained reasonably high (on par with or only slightly below the general model's sensitivity). In both Figures 6 and 7, the site‐specific QRF models were able to eliminate essentially all false positives on the test set (depending on the threshold), at the expense of flagging more plans overall (reducing sensitivity).

**FIGURE 6:**
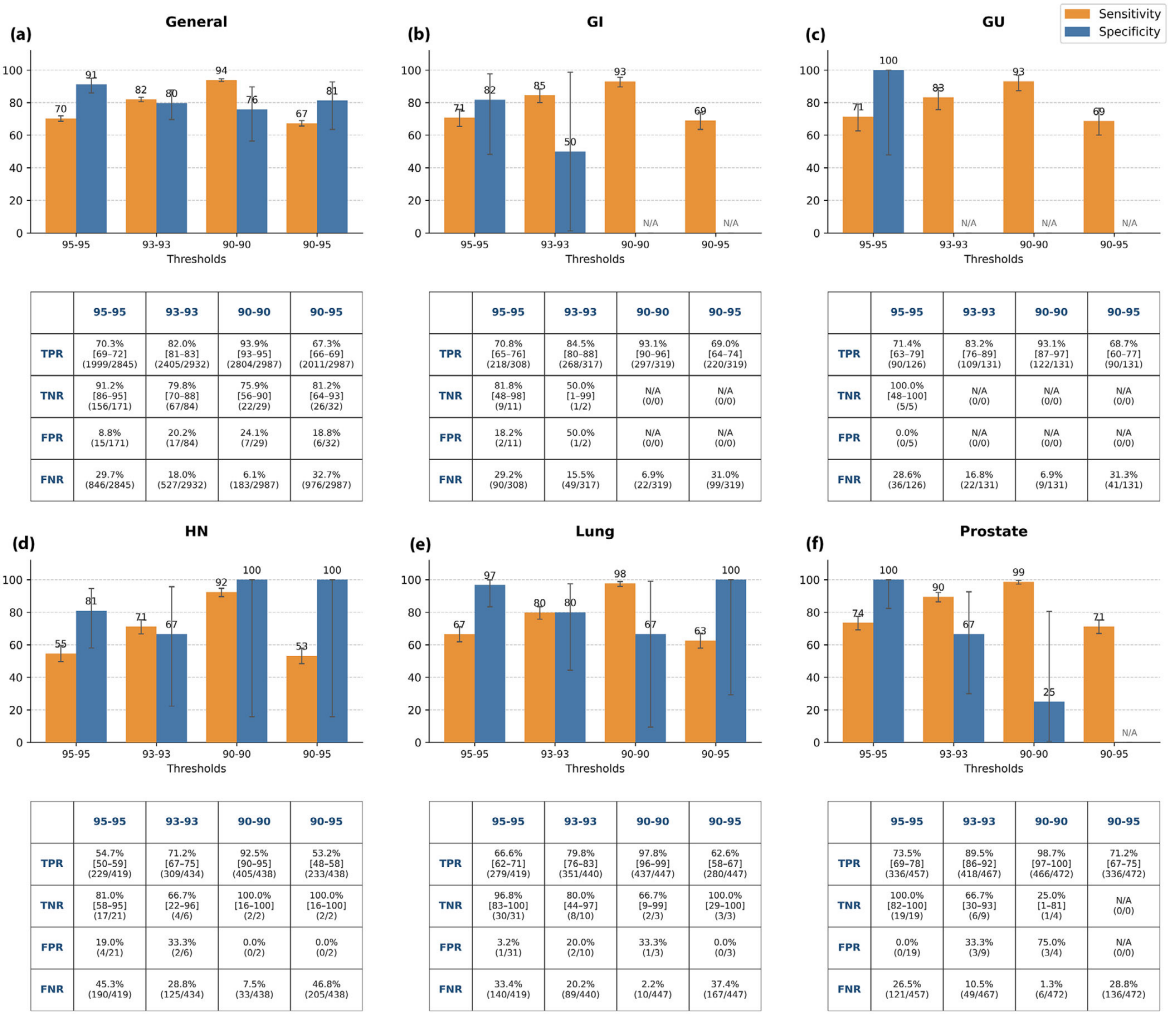
Sensitivity (also True Positive Rate) and specificity (also True Negative Rate) for the general and site‐specific QRF models for the various thresholds, with no flagging criteria. The true/false positive/negative rates (TPR, TNR, FPR, FNR) are outlined in the tables for each site. Sensitivity (TPR) and specificity (TNR) are each reported with their 95% Clopper–Pearson confidence intervals (shown in square brackets beneath the point estimate and count), and error bars on the bars indicate the same intervals. Cells with a zero denominator (no true failures at that threshold, i.e., 0/0) are marked “N/A” because specificity is undefined.

**FIGURE 7:**
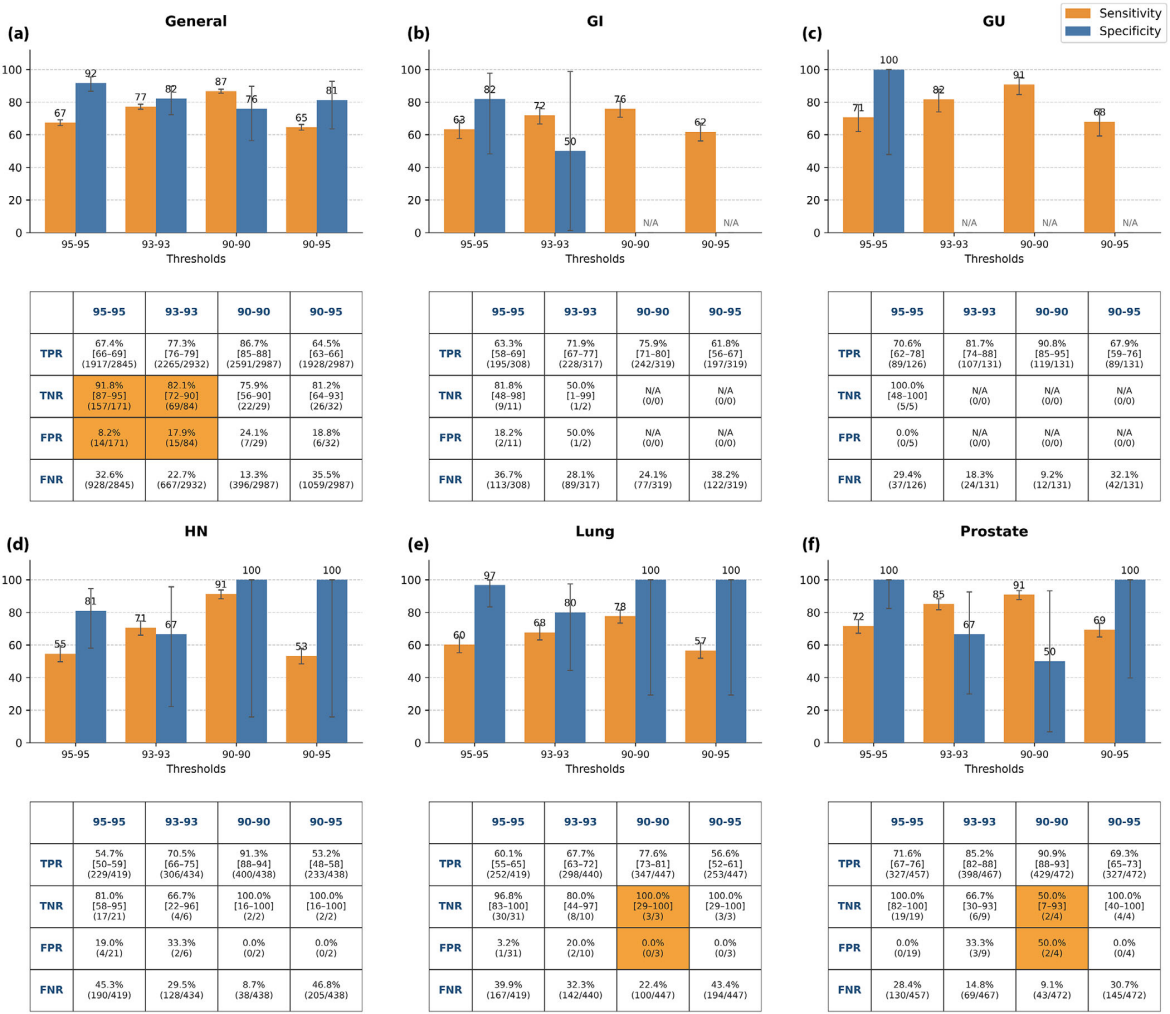
Sensitivity (also True Positive Rate) and specificity (also True Negative Rate) for the general and site‐specific QRF models for the various thresholds, with the flagging criteria as outlined in the Methods section. Plans are flagged as a ‘failing’ plan if they meet any one of the following criteria: GPR predicted as < the threshold, ALPO < 10, Total MU ≥ 2500, or MU Factor ≥ 10. Cells for which the specificity (TNR) is greater than the unflagged case and FPR is less than the unflagged case are highlighted in orange. Sensitivity (TPR) and specificity (TNR) are each reported with their 95% Clopper–Pearson confidence intervals (in square brackets beneath the point estimate and count), with matching error bars on the bars. Cells with a zero denominator (0/0, i.e., no true failures at that threshold) are marked “N/A” because specificity is undefined.

Figure 7 shows that the flagged mode slightly increases specificity in some cases, while sensitivity is slightly lower in most cases. Sensitivity in flagged mode dropped more noticeably in some cases—for instance, the GI and lung models’ sensitivity fell since many borderline plans were flagged. Despite the reduced sensitivity, this ultra‐high specificity mode is desirable in clinical practice where false positives are considered more costly than a slight increase in false negatives.

### 3.4 Learning curves

We quantified how prediction error scales with training set size using learning curves (Figure 8). For the general models, MAE plateaued after ∼964 training cases for QRF, versus ∼2000 for RFR and ∼4000 for XGB, indicating that ∼1000 diverse VMAT plans were sufficient to train a robust general QRF model in our setting. Site‐specific QRF models reached their performance plateau with fewer plans: GU at ∼80, HN at ∼320, GI at ∼480, lung at ∼640, and prostate at ∼960 training cases. The GU curve shows early diminishing returns, likely reflecting the relative homogeneity and lower complexity of GU plans at our institution (excluding prostate) and the dominance of kidney and bladder cases (Figure 1c), which are easier for the model to learn. By contrast, the prostate learning curve required nearly as many cases as the general model despite ultimately achieving a slightly lower MAE (0.83% vs. 0.92%). This larger data requirement is consistent with greater intrinsic variability: our prostate cohort includes prostate‐only, prostate + seminal vesicles, and prostate + seminal vesicles + nodes plans, and most prostate VMAT plans are SBRT with stringent rectal and bladder sparing objectives—factors that increase planning complexity and heterogeneity. Prior studies have trained prostate models on substantially smaller cohorts, but, to our knowledge, they did not systematically examine the number of training cases required to maintain model performance.^13^, ^17^, ^18^ Li *et al.* explored the impact of plan complexity parameters on measured GPR for different sites, and noted that MU and small apertures have a higher influence on prostate plans and prostate plans had the smallest aperture area compared with other plans (including HN).^19^

**FIGURE 8:**
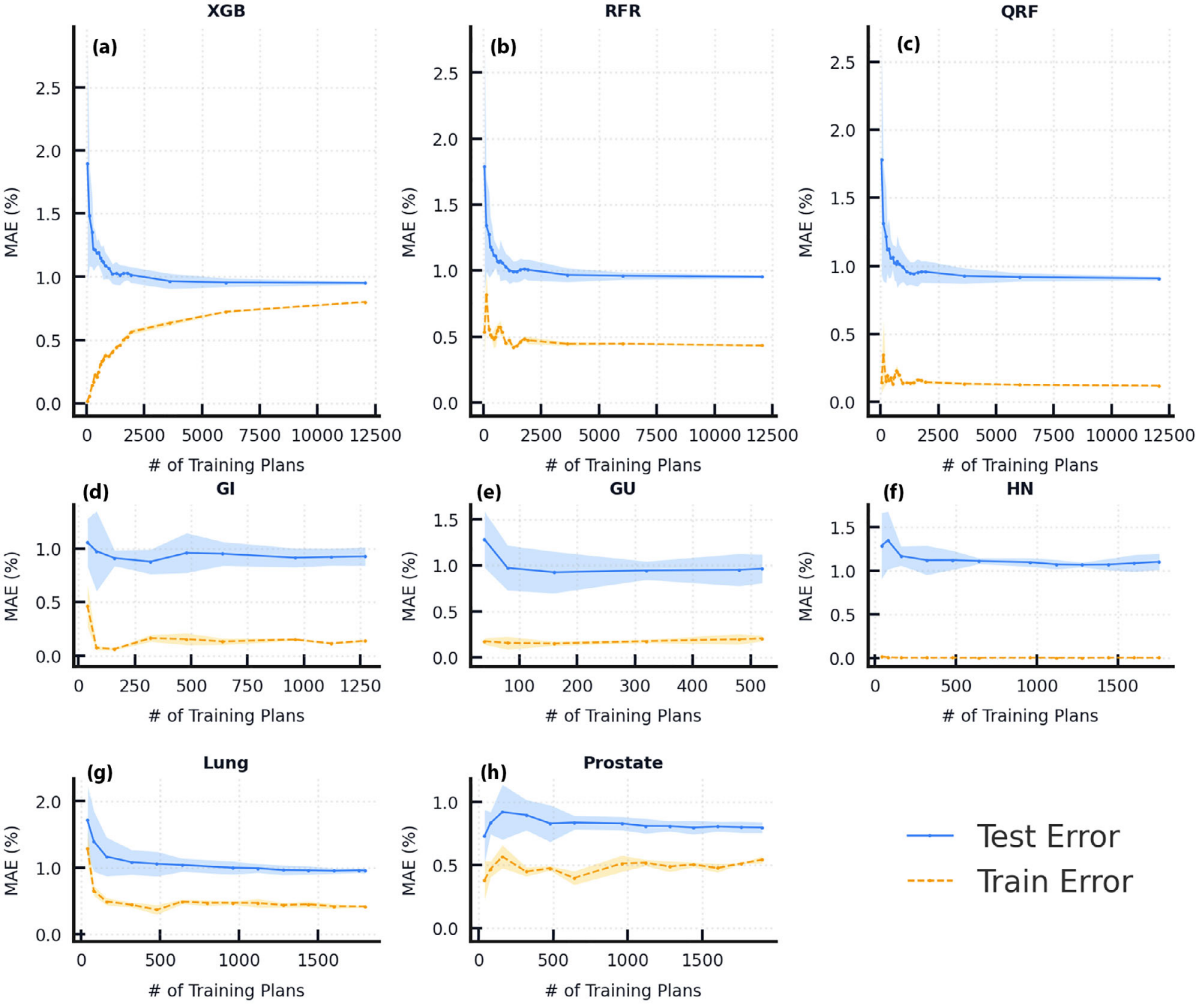
Learning curves for the general models (a–c) and the five site‐specific QRF models (d–h). Each curve shows the model's prediction error (in % mean absolute error or MAE) as a function of the number of training plans. For each model, training error (orange dotted line) and testing error (blue solid line) are plotted, illustrating how additional training data impacts performance. The shaded region for each curve represents the variability from 5‐fold cross‐validation as the standard deviation of MAE.

## 4 DISCUSSION

In this study, we developed a general PSQA outcome prediction model and compared it to site‐specific models for VMAT plans, demonstrating that tailoring the model to a particular disease site can substantially reduce false‐positive predictions. Our general model leveraged 15 000 VMAT PSQA measurements, one of the largest datasets used to train a model for virtual PSQA to date. The general QRF model's performance (MAE: 0.92% on 3%/3 mm GPR) is among the best reported in the literature, and the site‐specific models further improved specificity. This reflects two important insights: (1) plan complexity features drive gamma passing outcomes, and these features manifest differently across treatment sites, and (2) extremely high prediction accuracy (within ∼1%) is achievable with large datasets and appropriate model design. Our robust finding that MLC aperture complexity metrics (SAS at 20 and 10 mm, ALPO, etc.) are the top predictors of gamma passing rate, in all three general models tested (XGB, RFR, QRF), is consistent with previous studies.^2^, ^3^ In contrast to “black box” deep learning approaches, the feature importance from models like QRF offers transparency: clinicians can verify that the model is indeed sensitive to known predictors of delivery accuracy.

Critically, we showed that incorporating disease site information improves predictive performance. Prior studies have hinted at this need—Wall and Fontenot noted that treatment site was the third most important feature in their QA outcome model, suggesting that combining different sites in one model may introduce avoidable variability.^8^ Our work is the first to explicitly build and evaluate separate models per site. The site‐specific QRF models achieved equal or better accuracy than the general model and, more importantly, reduced false‐positive rates for certain sites. For example, the general model had a false‐positive rate of 8.8 % at the 95% threshold (flagging 15 failing plans as passes), whereas the prostate and GU models had *zero* false positives at the same threshold. High specificity strengthens confidence in virtual PSQA and is crucial for a virtual PSQA system to be implemented in the clinic. Our site‐specific models achieved close to 100% specificity (depending on GPR thresholds used), substantially higher than the ∼68%–91% specificity reported in prior studies that used general models.^1^, ^4^, ^17^

The benefit of site‐specific modeling was not uniform across sites, and head‐and‐neck (HN) is an exception. At the 95–95 operating point the HN site‐specific model reached only 54.7% sensitivity (229/419), which is lower than the general model's sensitivity for the same site (70.3%), and its specificity, although high, rested on a small number of true failures. In other words, isolating HN did not improve, and on the sensitivity axis worsened, performance relative to the pooled model. We believe this reflects the intrinsic heterogeneity of the HN site rather than a failure of the approach: HN plans at our institution span a wide range of prescription levels and target volumes and are typically the most highly modulated of all sites, producing a broader and lower GPR distribution with more borderline cases near the decision threshold. With this greater heterogeneity, a single‐site HN model does not benefit from the variance reduction that site isolation provides for more homogeneous sites such as GU or prostate, and it may in fact lose the stabilizing influence of the larger pooled dataset. We can infer from this case that site‐specific models are most advantageous for sites with relatively homogeneous, well‐behaved complexity distributions, whereas complex, heterogeneous sites such as HN may be served as well or better by the general model or may require additional features or larger site‐specific cohorts. An alternative to training fully separate per‐site models is to retain a single pooled model and add disease site as a categorical input feature, allowing the tree ensemble to partition on site internally where it is informative while still sharing statistical strength across sites. We did not pursue this design here because our primary aim was to test the hypothesis of whether completely isolating each site improves failure detection. Comparing pooled models that explicitly incorporate disease sites as an input feature with site models represents an important avenue for future work. It is important to balance specificity with sensitivity. In our results, all models showed a tendency for higher specificity than sensitivity, reflecting a conservative bias that is arguably appropriate in QA predictions (we prefer to err on the side of caution by flagging borderline plans). The general model's sensitivity (70% at 95–95) and the site models’ sensitivities (60%–90% range over all thresholds) are comparable to those reported by other authors for similar pass/fail criteria.^1^, ^4^, ^5^ Our approach using QRF lower quantile predictions, 90–95 threshold, and flagging criteria was our most conservative mode. Under this mode, we demonstrated that false positives can be virtually eliminated (100% specificity) for all site‐specific models, at the cost of reduced sensitivity (∼50%–70%). In practice, a clinic could choose an operating point on this spectrum based on tolerance for risk. Our general QRF model's sensitivity (70%) and the site models’ sensitivities (often > 70% and even approaching 90% in some cases) indicate a balanced performance where the majority of truly passing plans are correctly identified while minimizing false positives. It is worth noting that a recent generative adversarial network model by Matsuura *et al.* focused on predicting the entire gamma distribution for prostate plans and achieved very high specificity (99.6%) at the expense of sensitivity (∼51.9%) for 3%/2 mm tolerance.^18^ Our site‐specific QRF models, especially in flagged mode, achieve a similar high‐specificity regime without requiring deep learning and with the advantage that QRF provides uncertainty estimates and is easier to interpret and implement.

The examination of learning curves here informed how much data is needed to train reliable models. Machine learning in radiotherapy QA is often limited by the relatively small number of QA results available (compared to other big‐data domains). Many early studies used on the order of only a few hundred plans or even fewer per model. For instance, Tomori *et al.* trained a CNN on 60 prostate plans,^13^ but it was unclear how the model would scale with more data. In our analysis, the prostate model continued to improve until ∼900 cases. This suggests that had we built the prostate model on 60 cases, its error would have been higher. Groups that used ∼200–500 plans for training reported higher MAEs around 1.2%–2.5%.^2^, ^8^, ^17^, ^22^ Encouragingly, some sites require far less data: our GU model hit < 1% MAE with well under 100 training cases. This finding could guide institutions with smaller case volumes: for example, even a medium‐sized clinic could realistically collect 80 GU cases and train a preliminary model. On the other hand, for complex and diverse sites like HN, a larger sample (several hundred cases) is advisable to capture the full variability of plan configurations. The learning curve approach in our study provides a quantitative basis for deciding when additional data has diminishing returns, which is rarely addressed in prior QA prediction literature. We did not observe overfitting in any model once the curves plateaued—adding data consistently reduced both training and testing errors in tandem.

Although it is reasonable to consider whether the full approximately 15 000‐case dataset provided additional value, particularly given that the general QRF model's MAE appeared to plateau after roughly 964 cases and several site‐specific models plateaued with fewer cases, the MAE learning‐curve plateau should not be interpreted as the point beyond which additional data are no longer informative. The learning curves track average regression error (MAE), a metric that, as noted above, is dominated by the large majority of high‐GPR passing plans and saturates quickly once those are well modeled. The plateau therefore indicates that mean accuracy stops improving, not that the clinically important behavior—detection of the rare failing plans and the stability of site‐specific specificity estimates—has stopped improving. The larger dataset contributed in three concrete ways that the MAE curve does not capture. First, it enabled the site‐specific analysis itself: only because thousands of plans were available could we assemble per‐site cohorts large enough to train and, more importantly, to evaluate separate models. Second, it increased the absolute number of failing plans available for both training and testing; because failures are so rare (∼5.7%), even a modest test set yields only a handful of true negatives per site, and the wide confidence intervals reported in Figures 5–7 show directly that specificity estimates remain statistics‐limited even at this dataset size. A smaller dataset would have produced even fewer failures and correspondingly less reliable specificity estimates. Third, the larger sample improves robustness to the heterogeneity of real clinical practice over a multi‐year period. Beyond the plateau, most additional plans are high‐GPR passing cases that contribute little to the regression error, and the marginal value of further data lies primarily in accumulating failing cases and in extending the approach to additional sites rather than in reducing mean MAE.

### 4.1 Clinical implications

The high specificity achieved by site‐specific QRF models means that a virtual PSQA system could reduce the number of measurement‐based QAs without compromising safety. For example, using the prostate‐specific model with a 95% threshold, none of the prostate plans with measured GPR < 95% went undetected in our test set (0 false positives, specificity 100%, 19/19). It is important to note, however, that this point estimate rests on a small number of true failures and carries a wide 95% confidence interval (82.4%–100%, Clopper–Pearson); the data are therefore consistent with a true false positive rate of up to roughly 18%. Previous prospective studies of “virtual QA” have reported the potential to cut the QA workload by 60%–70%.^4^, ^6^ Our findings with an even more targeted reduction when site‐specific models are employed, with flagging criteria helping to identify plans that should be measured. Furthermore, by deploying site‐specific models, physics teams can develop site‐specific flagging criteria. For instance, if the HN model flags a plan, it may be due to extremely high modulation or small apertures that are unusual for typical HN plans; recognizing this can help in plan review or in providing feedback to planners. Over time, this feedback loop could improve planning practices for each disease site (an aspect of quality assurance optimization (QAO) as described by Wall *et al.* ^23^).

### 4.2 Limitations and future work

This study is based on data from a single institution using one QA device (ArcCheck) and a single gamma analysis criteria (3%/3 mm global). While the dataset is large and spans multiple years and machines, our model performances cannot be generalized to other institutions or different tolerances. Multi‐center evaluations (e.g., Lambri *et al.* ^1^) have shown that models trained on one center's data can degrade in performance when applied elsewhere, likely due to differences in machines, patient populations, and planning techniques. A logical extension of our work would be to test *multi‐institution site‐specific models*—for example, pooling prostate plan data from several clinics to see if a robust prostate model can generalize across centers. Another limitation is that we only built site models for the five most common sites in our dataset. In the future, as more data accumulate for other sites (such as gynecological and brain), separate models could be trained or transfer learning applied from similar sites (e.g., leveraging the prostate model for other pelvic sites). We also did not explicitly model the effect of QA device measurement noise or uncertainties in gamma calculation. The QRF's quantile output partly addresses this by indicating a range of likely outcomes.

A further consideration concerns the temporal span of the data. The cohort was acquired over 2013–2024 across three Pinnacle versions and an evolving clinical practice, and our random 80/20 train/test split mixes plans from across this interval, so training and test sets are drawn from the same time‐mixed distribution. We therefore examined the data directly for temporal drift and found none: neither measured nor predicted GPR, nor plan complexity, changed significantly over the study period, either overall or within individual disease sites (Figures S1 and S2). This stability supports pooling plans across years and suggests our institution's planning and QA practice was sufficiently consistent that the time‐mixed split does not bias the evaluation. A complementary and more stringent check would use a temporally held‐out test set (for example, training on earlier years and testing on the most recent year) to assess how well performance generalizes forward to the most recent practice period; we regard this as useful future work. Additionally, the empirical flagging thresholds (ALPO < 10, total MU ≥ 2500, MU factor ≥ 10) were derived from our institution's data and would need to be re‐derived locally before use elsewhere. A particularly important limitation is the exclusive use of the 3%/3 mm global gamma criterion at our institution. AAPM TG‐218 has encouraged the field toward tighter tolerances—3%/2 mm, and 2%/2 mm for stereotactic deliveries—under which measured failure rates are typically substantially higher than at 3%/3 mm. Our conclusions about specificity and the feasibility of omitting measurements are therefore specific to the relatively permissive 3%/3 mm criterion and cannot carry over unchanged to tighter criteria. We expect two competing effects under tighter tolerances. On one hand, the larger minority (failing) class would directly mitigate the statistical fragility: with more true failures per site, specificity estimates would rest on larger denominators and yield substantially narrower confidence intervals, making any high‐specificity claim more robust than the small‐denominator estimates reported here. On the other hand, the prediction problem itself becomes harder, because GPRs at 3%/2 mm and 2%/2 mm are lower, more dispersed, and more sensitive to delivery and modeling details, so absolute prediction error tends to grow and the separation between passing and failing plans narrows. The net effect on achievable sensitivity and specificity is not obvious a priori and likely varies by site, with the most highly modulated sites (e.g., HN) and stereotactic prostate plans expected to be the most challenging. Re‐deriving the site‐specific models and operating points under 3%/2 mm and 2%/2 mm criteria is a clear priority for future work and would more directly address the safety‐critical, low‐failure‐rate regime relevant to clinical implementation.

Finally, while many prior works have pursued increasingly complex algorithms (from support vector machines^23^ to deep neural networks^13^, ^15^), our results demonstrate that tree‐based ensemble models remain extremely effective. The QRF in particular strikes a valuable balance: It provides prediction uncertainty, is interpretable via feature importance, and yielded performance on par with state‐of‐the‐art methods. The clinical deployment of such models is also relatively straightforward; in fact, to the best of our knowledge, only one group has reported moving their PSQA prediction models into real clinical workflow to date.^4^, ^6^ Witztum *et al.* integrated an XGBoost model for VMAT PSQA prospectively over 2 years, achieving 100% specificity and 67% sensitivity.^6^ Our work adds to this momentum by providing a refined approach that could further reduce unnecessary QA checks. The next step will be to prospectively validate our site‐specific QRF models within our clinic's workflow. We plan to perform a phased implementation where the model recommendations (whether to skip or proceed with measurement) are recorded and compared against actual QA outcomes in real time, similar to what has been done by Witztum *et al.* This will allow us to assess prospective accuracy and confidence in the model. Adopting site‐specific models for VMAT QA could save substantial time and resources, enabling physicists to focus attention on the plans that truly need intervention while safely forgoing measurements on those predicted to pass. Ultimately, this approach aligns with the broader trend of utilizing machine learning to enhance quality assurance efficiency and could be extended to other complex treatments (e.g. IMRT, SRS) and other QA devices or metrics.

## 5 CONCLUSION

This work rigorously compared general and site‐specific virtual PSQA models for VMAT plans, demonstrating that a site‐specific approach substantially improves predictive performance and is optimal for clinical deployment. Using a Quantile Random Forest (QRF) model on a large dataset of 15 000 PSQA measurements, the general model achieved high accuracy (MAE: 0.92%), and the tailored site‐specific models substantially reduced false‐positive predictions; most notably, the prostate and GU models achieved 100% specificity (zero false positives) at the 95% GPR threshold. High specificity is an important prerequisite for clinical confidence in virtual QA workflows, as it supports the selective omission of physical QA measurements for low‐risk plans and may enable meaningful reductions in measurement workload and resource utilization. Furthermore, our analysis of learning curves provides a quantitative guide on data requirements, showing that while complex sites need hundreds of cases, simpler sites like GU can be modeled robustly with fewer than 100 training cases, facilitating adoption even by smaller clinics. This work demonstrates that an interpretable model like QRF, when tailored to the site, can achieve performance comparable to advanced deep learning methods, offering a straightforward path toward clinical integration. The next logical step is the prospective validation of these site‐specific models within the clinical workflow to realize their full efficiency‐maximizing potential.

## AUTHOR CONTRIBUTIONS

*Conceptualization and Methodology*: Dylan Yamabe Breitkreutz, Greg Kaliel, Mark D'Souza and Naila Rahman. *Data Collection*: Dylan Yamabe Breitkreutz, Greg Kaliel and Naila Rahman. *Data Analysis*: Dylan Yamabe Breitkreutz, Greg Kaliel and Naila Rahman. *Manuscript Drafting*: Naila Rahman. *Edits and Final Approval of Manuscript*: Dylan Yamabe Breitkreutz, Greg Kaliel, Mark D'Souza and Naila Rahman.

## CONFLICT OF INTEREST STATEMENT

The authors declare no conflicts of interest.

## ETHICAL APPROVAL

No ethics approval was required by our research ethics board for this work.

## Supporting information



Supporting Information

## Data Availability

The data supporting the findings of this study are available from the corresponding author upon reasonable request.
