# Supplementary material for: General versus site specific quantile random forest models for virtual patient‐specific quality assurance of VMAT plans
Source: J Appl Clin Med Phys. 2026 Oct 16;27(10):e70854. doi: 10.1002/acm2.70854 (PMC13673225; doi:10.1002/acm2.70854)
Supplement: Supplementary file 1 [file ACM2-27-e70854-s001.pdf]

Supplementary Material

	RFR (general)	QRF (general)	GI	GU	HN	Lung	Prostate
Bootstrap	False	True	True	True	False	True	True
max_features	sqrt	log2	log2	log2	sqrt	sqrt	sqrt
min_samples_leaf	4	1	2	1	1	1	1
max_depth	30	15	25	25	25	20	10
min_samples_split	2	8	4	5	5	10	10
n_estimators	350	450	350	650	500	500	475

Supplementary Table 1. *Hyperparameters for each model. Hyperparameters are listed for the RFR and QRF general models, as well as the QRF site-specific models. For the general XGB model, the hyperparameters were: alpha = 0.1; colsample_bytree = 0.8; learning_rate = 0.05; max_depth = 5; min_child_weight = 1; n_estimators = 200; subsample = 1.*

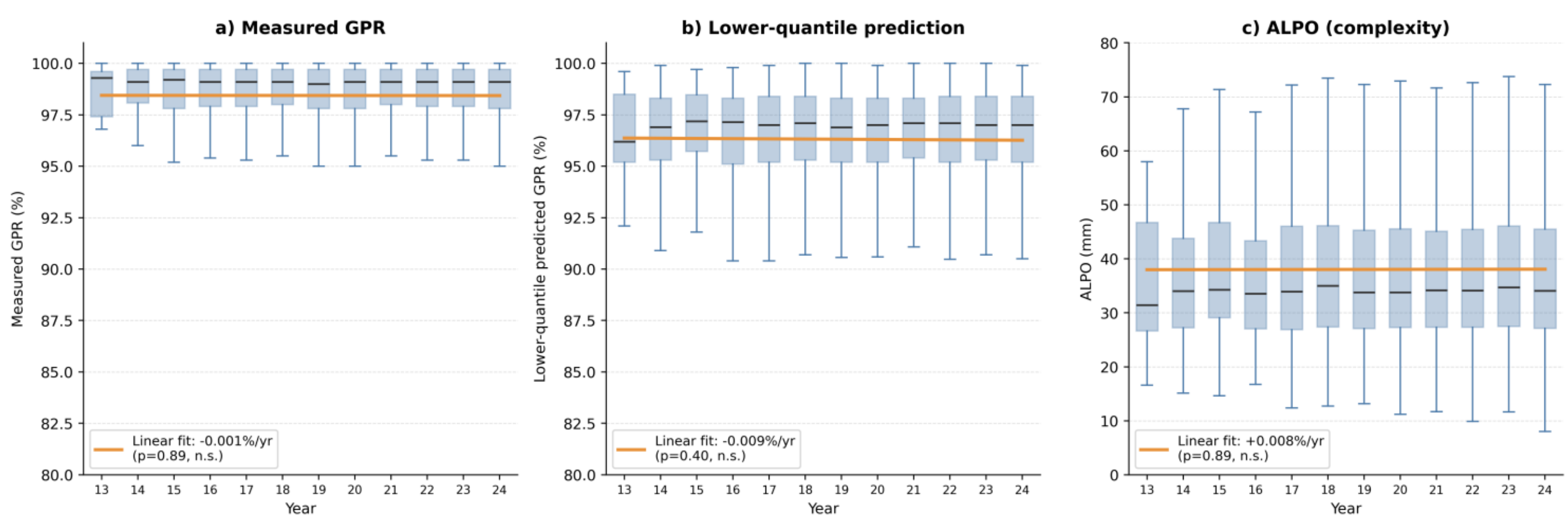

Supplementary Figure 1. Temporal stability of measured and predicted gamma passing rate (GPR) and plan complexity, 2013–2024. Box plots show the yearly distribution of (a) measured GPR, (b) the QRF lower-quantile predicted GPR, and (c) ALPO, an aperture-based plan-complexity metric. Boxes span the interquartile range with the median indicated; whiskers extend to 1.5× the interquartile range. The orange line in each panel is the

ordinary-least-squares linear fit across all plans, with its slope (per year) and p-value annotated; none of the trends differed significantly from zero.

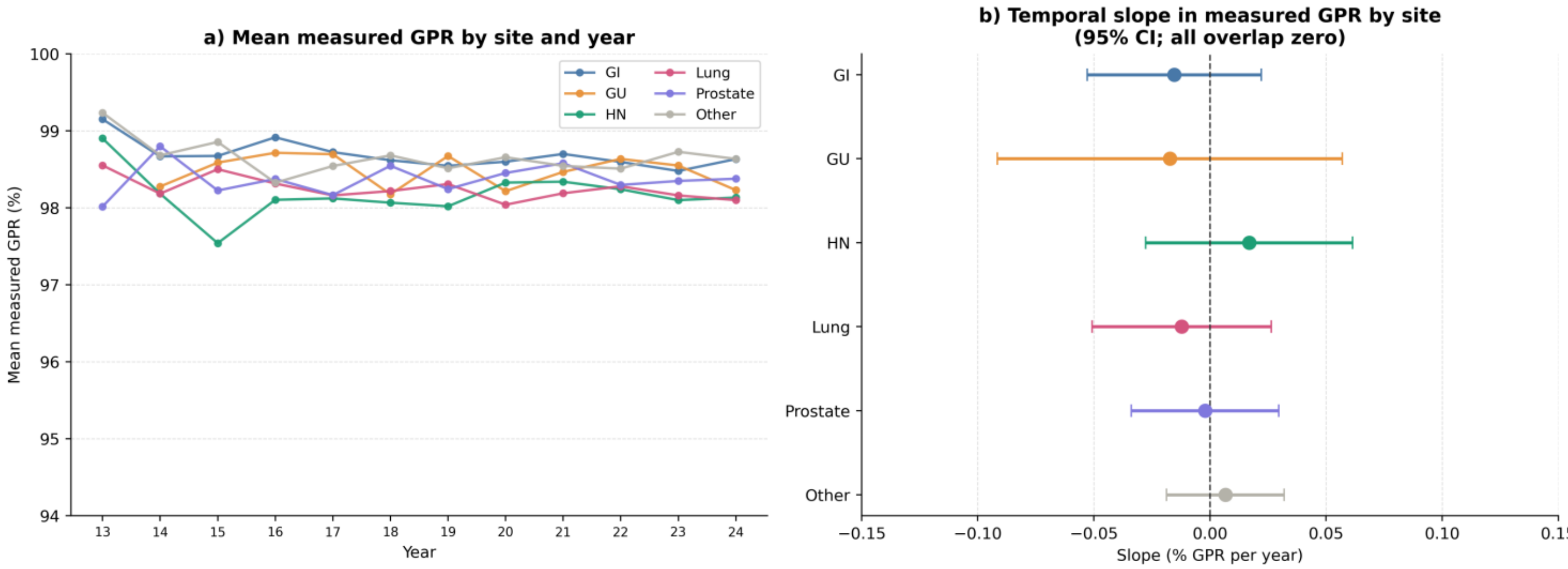

Supplementary Figure 2. Absence of temporal drift within individual disease sites. (a) Mean measured GPR by treatment year for each disease site, showing all sites remaining within a narrow ~1% band across the study period. (b) Forest plot of the site-specific temporal trend in measured GPR: each point is the ordinary-least-squares slope (change in GPR per year) for one site, with horizontal bars indicating the 95% confidence interval. All confidence intervals cross zero (dashed line), indicating no statistically significant temporal drift in any site.

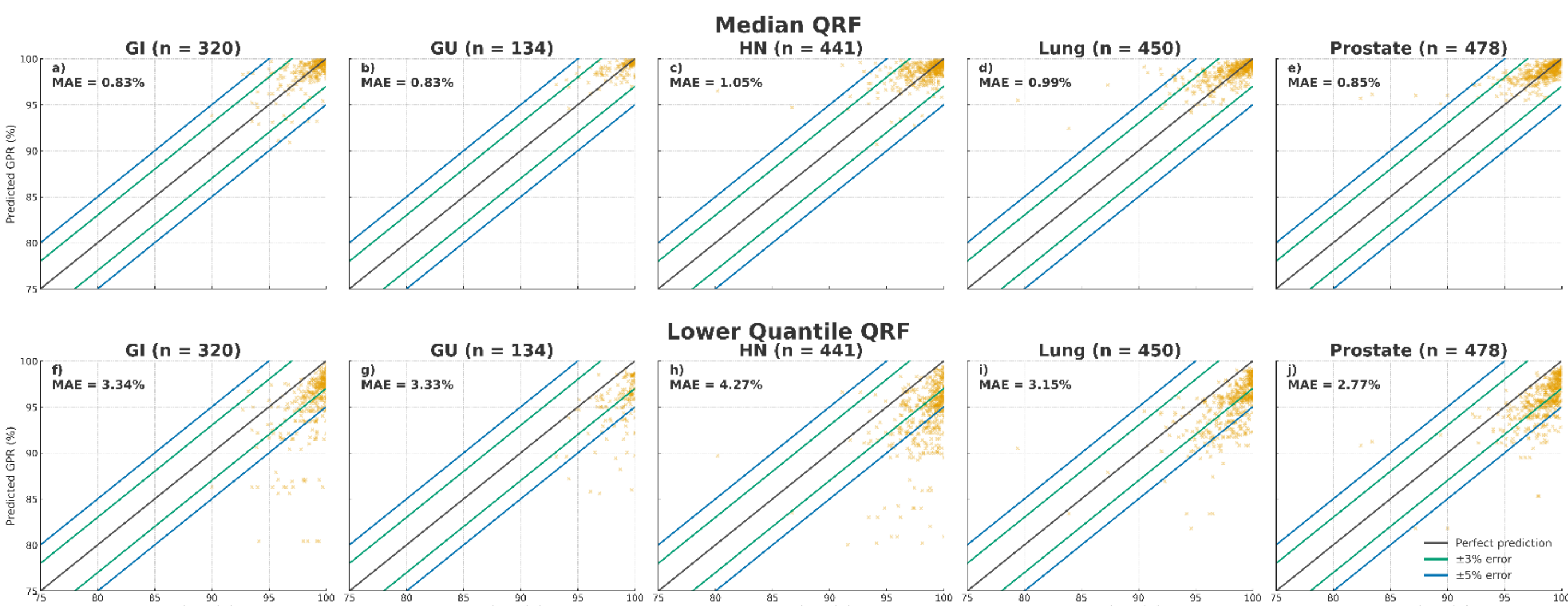

Supplementary Figure 3. *Distribution of true (measured) and predicted gamma passing rate (GPR). Median QRF predictions (top row, panels* ***a – e****) and the lower quantiles (bottom row, panels* ***f – j****) are shown across all sites.*

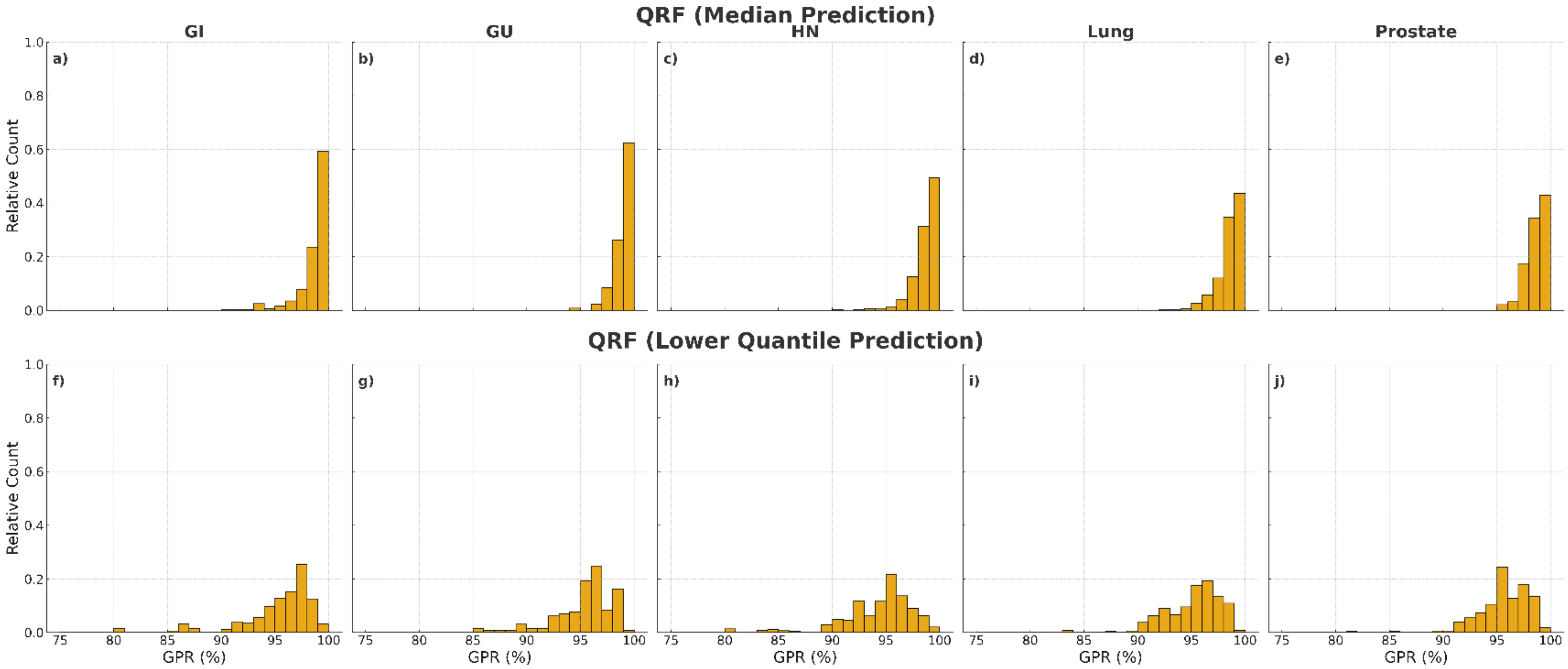

Supplementary Figure 4. Distributions of site-specific GPRs predicted by the QRF model. **Top row (a-e):** Histograms for median predictions from each site-specific QRF model. **Bottom row (f-j):** Histograms for lower quantile predictions from each site-specific QRF model. Histograms use 1-percentage-point bins over 75–100% GPR.
